# Accuracy of Fall Prediction in Parkinson Disease: Six-Month and 12-Month Prospective Analyses

**DOI:** 10.1155/2012/237673

**Published:** 2011-11-30

**Authors:** Ryan P. Duncan, Abigail L. Leddy, James T. Cavanaugh, Leland E. Dibble, Terry D. Ellis, Matthew P. Ford, K. Bo Foreman, Gammon M. Earhart

**Affiliations:** ^1^Program in Physical Therapy, Washington University in St. Louis School of Medicine, St. Louis, MO 63108, USA; ^2^Department of Physical Therapy, University of New England, Portland, ME 04103, USA; ^3^Department of Physical Therapy, University of Utah, Salt Lake City, UT 84108, USA; ^4^Department of Physical Therapy and Athletic Training, Boston University, Boston, MA 02215, USA; ^5^Department of Physical Therapy, University of Alabama at Birmingham School of Health Professions, Birmingham, AL 35294, USA; ^6^Department of Anatomy & Neurobiology, Washington University in St. Louis School of Medicine, St. Louis, MO 63110, USA; ^7^Department of Neurology, Washington University in St. Louis School of Medicine, St. Louis, MO 63110, USA

## Abstract

*Introduction*. We analyzed the ability of four balance assessments to predict falls in people with Parkinson Disease (PD) prospectively over six and 12 months. *Materials and Methods*. The BESTest, Mini-BESTest, Functional Gait Assessment (FGA), and Berg Balance Scale (BBS) were administered to 80 participants with idiopathic PD at baseline. Falls were then tracked for 12 months. Ability of each test to predict falls at six and 12 months was assessed using ROC curves and likelihood ratios (LR). *Results*. Twenty-seven percent of the sample had fallen at six months, and 32% of the sample had fallen at 12 months. At six months, areas under the ROC curve (AUC) for the tests ranged from 0.8 (FGA) to 0.89 (BESTest) with LR+ of 3.4 (FGA) to 5.8 (BESTest). At 12 months, AUCs ranged from 0.68 (BESTest, BBS) to 0.77 (Mini-BESTest) with LR+ of 1.8 (BESTest) to 2.4 (BBS, FGA). *Discussion*. The various balance tests were effective in predicting falls at six months. All tests were relatively ineffective at 12 months. *Conclusion*. This pilot study suggests that people with PD should be assessed biannually for fall risk.

## 1. Introduction

Postural instability is a common cause of falls in people with Parkinson disease (PD) [1]. In contrast to community-dwelling adults over age 65, approximately one-third of whom report falling each year [2], up to 70% of individuals with PD fall once annually, while 50% fall twice or more in a one year period [3, 4]. Falls lead to a myriad of complications [5] that can affect not only physical health, but also the psychological health of the individual. Hip fracture and head trauma are two of the most common physical problems incurred by an individual with PD following a fall [6], while the psychological complications include fear of falling [7, 8] and reduced quality of life [9]. Such fall-related complications are associated with substantial economic costs [10, 11] and indicate an urgent need to identify and protect those individuals at the greatest risk.

Despite the relatively high prevalence of falls in the PD population, accurate and useful methods for predicting an impending future fall, especially during the early stages of the disease, remain elusive. Fall history, a well-known fall risk factor among older adults [12], has a limited utility as a solitary predictive indicator. Although a meta-analysis of prospective studies of falling in PD found that 57% of individuals who had a history of falls in the past year fell during a 3-month surveillance period, so did 21% of individuals with no history of falls [13]. Moreover, fall incidence alone does not help to identify underlying contributors to postural instability specific to PD. People with PD, for example, may demonstrate impairments in areas of movement control such as sensory integration, keeping their center of mass within their base of support, coordination of anticipatory postural control tasks [8, 14] as well as medication side effects such as dyskinesias [15]. For this reason, standardized balance assessment tools have been recommended to help determine factors contributing to falls so that therapeutic intervention targets can be identified [16, 17].

 The utility of a variety of clinical balance tests has been studied. Balance assessments including the Tinetti [18], Berg Balance Scale (BBS) [19], the Timed Up and Go (TUG) [20], the Functional Gait Assessment (FGA) [21], and recently developed Balance Evaluation Systems Test (BESTest) [22] have been shown to have sensitivity and specificity that exceeds a random guess, but they still demonstrate a clinically relevant proportion of false-positive and false-negative predictions [5, 23]. As noted in a previous meta-analysis [13], new prediction methods are needed. Relatively newly developed balance assessments such as the Functional Gait Assessment (FGA) [21], BESTest [22], and Mini-BESTest, a condensed version of the BESTest [24], have yet to be studied and compared prospectively.

Regardless of the balance assessment utilized, there have been efforts to improve the predictive performance on these balance assessments through diagnosis-specific alterations of cutoff scores or collective interpretation of multiple tests [15, 23, 25, 26]. While these methods may improve accuracy, their overall success may be limited by participant's fall recall bias. To date, we are unaware of any studies that have examined and compared whether the length of prospective follow-up affects the accuracy of fall prediction in persons with PD.

In order to address these gaps in our understanding of fall prediction in persons with PD, the primary objective of this study was to compare the relative accuracy for fall prediction of four common balance assessments at the six-month and 12-month prospective time points. Relative to our primary objective, we hypothesized that these tests would be useful in predicting falls prospectively at both six and 12 months, with better accuracy over the shortest of the two time periods. Our secondary objective was to compare the predictive accuracy and the validity indices of the four balance assessments. Relative to our secondary objective, we hypothesized that tests such as the FGA, BESTest, and Mini-BESTest that incorporate dynamic tasks would demonstrate improved predictive ability compared to the BBS.

## 2. Methods

### 2.1. Participants

We recruited participants using contact information gathered from the Washington University School of Medicine's Movement Disorders Center database and the Volunteers for Health database. Participants were recruited as part of a larger study [27]. Individuals were included if they had a medical diagnosis of idiopathic PD (Hoehn and Yahr (H&Y) Stages I–IV), were over the age of 40 and were community dwellers. Study candidates were excluded if they had atypical parkinsonism or previous surgical management of PD (pallidotomy or deep brain stimulation). Prior to participation, each participant provided written informed consent in accordance with the policies and procedures of Washington University School of Medicine's Human Research Protection Office.

### 2.2. Data Collection

Participants were evaluated at baseline utilizing four balance tests (BBS, FGA, BESTest, and Mini-BESTest) as described below under Balance Assessments. Participants were then followed for 12 months, with fall incidence determined through participant's report at the six-month and 12-month time points. An individual was considered a faller if he or she reported two or more falls over the surveillance period of interest (0–6 months or 0–12 months). An individual was considered a nonfaller if he or she reported zero or one falls during the surveillance period.

### 2.3. Balance Assessments

The BBS is a well-established balance measure consisting of 14 items (sit to stand, transfers, forward reach, etc.) used to determine whether or not one may be at risk for falls [28]. The BBS does not evaluate the balance during walking. It has been shown to be reliable when used to assess balance in people with PD [29]. Each item is scored on a scale of zero (indicating impaired balance) to four (indicating no impairment in balance), with a maximum possible score of 56.

The FGA [21] is a 10-item test of dynamic balance in which all components are evaluated while the participant is walking. Items performed by the participant include forward and backward walking as well as walking while turning the head, changing walking speeds, stepping over obstacles, and walking with a narrow base of support. When used to evaluate individuals with PD, this test had high interrater and test-retest reliability [23]. Each item is scored on a scale of zero (indicating loss of balance, increased time to perform task, significantly altered gait pattern) to three (indicating no impairment of gait or balance and completion of the task in a timely manner), with a maximum possible score of 30.

The BESTest [22] is a measure designed to evaluate balance control via 36 items that are divided into six sections (biomechanical constraints, stability limits and verticality, anticipatory postural adjustments, postural responses, sensory orientation, and stability in gait). Items in the BESTest include selected items from the aforementioned assessments (i.e., BBS and FGA) as well as items such as center of mass alignment, hip and ankle strength, sitting verticality and lateral lean, and multidirectional compensatory stepping correction, among others. The BESTest has high interrater and test-retest reliability in PD [23]. Each item is scored on a scale of zero (indicating poor balance or inability to complete task) to three (no impairment in balance), with a maximum score of 108 points.

A shortened version of the BESTest, the Mini-BESTest, was designed “to improve the structure and measurement qualities” of the BESTest [24]. This shorter version can be administered more quickly than the full BESTest, thereby reducing clinician and patient burden. The Mini-BESTest is a 14-item balance evaluation that concentrates on dynamic balance and its components are derived from four of the six BESTest sections. Items are scored on a scale of zero (poor balance) to two (no impairment of balance), with a maximum possible score of 32 as two of the 14 items receive two separate scores for different aspects of the tasks [30].

### 2.4. Procedures

All balance assessments were administered in the Locomotor Control Laboratory at Washington University School of Medicine by a trained physical therapist. Baseline assessments of participants began in July and ended in December of 2009. All participants maintained their normal medication regimen so that they were tested in the “on” phase of their medication, one to two hours after medication intake. Demographic information, fall incidence, and Movement Disorder Society Unified Parkinson Disease Rating Scale Motor Subscale III (MDS-UPDRS-III) scores were obtained prior to administration of balance assessments [31, 32]. Regarding fall incidence, participants were followed prospectively and at six months reported how many times they fell in the period from baseline to six months. At 12 months, participants reported how many times they fell in the period between six and 12 months, with number of falls from 0 to 12 months determined by adding the two reports together. Participants chose from the following answers: (1) none, (2) one time, (3) 2–10 times, (4) weekly, or (5) daily. An individual was classified as a faller if he experienced two or more falls in the period of interest (i.e., from baseline to six months or from baseline to 12 months). The order of balance assessments was as follows: BBS, FGA, and BESTest. Mini-BESTest scores were derived from the BESTest item scores, as all items on the Mini-BESTest are included in the BESTest. Items that were duplicated among the BBS, FGA, and BESTest were performed only once and scored appropriately for each tool. For example, a sit-to-stand transfer task is in the BBS and BESTest; therefore it was only performed once and scored by the rater according to the criteria listed on each tool.

### 2.5. Data Analysis

In order to test our primary hypothesis, receiver operating characteristic curves (ROCs) were constructed for each balance assessment at each time point (six and 12 months) and the area under the curve (AUC) was determined for each test at each time point. Using previously established cutoff scores [23], we determined the area under the curve (AUC), positive and negative likelihood ratios, and posttest probabilities for each test at each time point [33–35]. The time point that consistently produced the balance assessments with higher AUC and positive LR as well as lower negative LR would be interpreted as the more accurate time point. Once that determination was made, we examined our secondary objective and hypothesis through the use of empirical tests for noninferiority that were used to make pairwise comparisons of the AUC for each test (*P* < 0.05) [36]. Point estimators and interval estimators (95% confidence intervals [95% CI]) were calculated for all AUC and likelihood ratio values.

## 3. Results

Baseline evaluations were completed on 80 participants. Of the original cohort, 51 participants (41% male) completed the six-month evaluation, and 40 participants (40% male) completed the 12-month evaluation (Table 1). At six months, 14 individuals (27%) were considered fallers, while 13 individuals (32%) were considered fallers at 12 months. Regarding reasons for dropout at six months, 15 participants were unable to be contacted or gave no reason for discontinuing, nine experienced a decline in condition or an unrelated medical condition, one had transportation difficulty, one participant experienced family problems, and three participants had incomplete data sets. At 12 months, in addition to those who had dropped out by six months, four participants were unable to be contacted or gave no reason for discontinuing, three experienced a decline in condition or an unrelated medical condition, and four participants had incomplete data sets. Of the 11 individuals that were lost from six to 12 months, seven (three males) were characterized as fallers at six months.

### 3.1. Comparison of Six- and 12-Month Results

At six months, AUCs for the tests ranged from 0.8 to 0.89, while at 12 months, AUCs ranged from 0.68 to 0.77. At six months (Table 2(a)), the positive likelihood ratios were greater, the negative likelihood ratios were lower, and the posttest probability values were lower (i.e., better) for all for balance tests than at 12 months (Table 2(b)).

### 3.2. Individual Test Comparison

**Figure 1:**
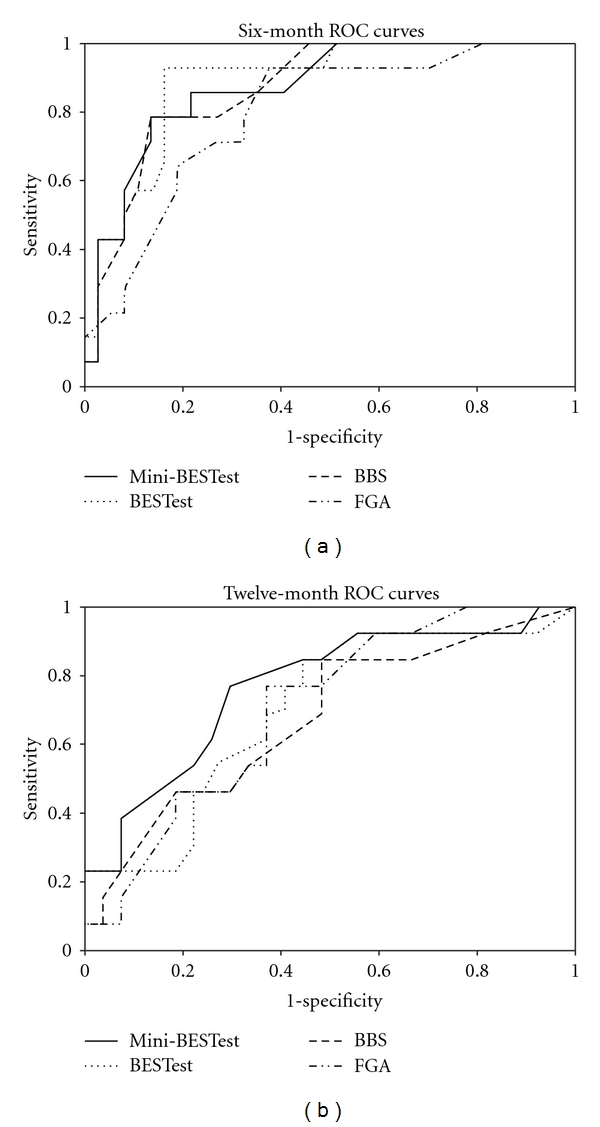


Based on the apparent greater accuracy of the six-month prediction, the individual tests were compared at the six-month time point to determine which, if any, was superior to the others in terms of predictive ability. All tests provided greater accuracy than a random guess, with AUC point estimators ranging from 0.89 (BESTest) to 0.80 (FGA) and substantially overlapping 95% CIs (Table 2(a), Figure 1). However, noninferiority tests revealed that the AUC of the BESTest was superior to that of all other tests. Noninferiority tests also showed that the FGA was inferior to all other tests (Table 3).

## 4. Discussion

Previous prospective studies of fall prediction have utilized varied lengths of follow-up period [5, 13]. However, to our knowledge, no previous work has directly compared the accuracy of fall prediction at different follow-up intervals. Our data confirmed our primary hypothesis that a shorter follow-up period (six months) consistently produced more accurate predictions than a longer follow-up period (12 months). In addition, at the six-month follow-up time point, all of the balance assessments studied provided clinically useful predictive accuracy. Comparisons of the point estimators and statistical tests of noninferiority suggested that the BESTest produced the greatest predictive accuracy. However, it is unclear whether the differences between the BESTest and the other balance measures are sufficiently large to merit use of one test over another in a clinical setting.

### 4.1. When Should Fall-Related Screening Take Place?

The recently published American Academy of Neurology quality of care measures for Parkinson Disease state that persons with PD should be assessed for fall-related issues “at least annually [37].” While these guidelines provide targets for clinicians, they were developed through a consensus building process that involved expert panel input, public comment, and stakeholder input, and therefore lacked research-based support. Our findings of both six- and 12-month predictive accuracy having AUC values greater than 0.50 (the level of a random guess) support this metric. However, if clinicians wish to most accurately assess the risk of falling of a person with PD, our data suggest that they should consider that biannual follow-up of persons with PD regarding falls.

### 4.2. Is One Test Better Than Another?

The validity indices (AUC, positive and negative likelihood ratios) demonstrated that all of the tests studied provided clinically meaningful predictive ability. Substantial overlap of the interval estimators agreed with previous studies that have documented moderate levels of accuracy for the BBS and the FGA [16, 23]. In this sample, the point estimators of the validity indices and the tests for noninferiority indicated that the BESTest provided the highest level of accuracy and, for the first time, provided prospective documentation of its predictive validity. The BESTest's likelihood ratio modifications to the pretest probability of being a faller provide a specific example of the clinical relevance of these findings. At six months, the pretest probability of being a faller was 27%. Based on the BESTest positive likelihood ratio, an individual who scored below the cutoffs for the BESTest increased their posttest probability of being a faller to 69%. Based on the BESTest negative likelihood ratio, an individual with a score above the cutoff reduced their posttest probability of being a faller to 3%. These modifications to the pretest probability are similar to those observed in other studies of persons with PD [25].

While our results suggested that the BESTest may be the most accurate as a free-standing test to predict falls in the absence of other balance assessments, the administration time of the BESTest is much longer than the other three assessments. Although the results of this study support its use when assessing balance and fall risk in individuals with PD, it is not clear whether the slightly improved accuracy of the BESTest as compared to the Mini-BESTest or BBS at six months is enough to merit utilization of the full BESTest in clinical settings where time constraints must be considered.

We found it surprising that the BESTest, Mini-BESTest, and BBS outperformed the FGA when used prospectively over six months. Based on previous research, we hypothesized that more dynamic balance tests such as the BESTest, Mini-BESTest, and FGA would be more likely to accurately predict falls than a less dynamic balance test like the BBS [23, 38]. However, our findings regarding this hypothesis were mixed, with the FGA having the lowest predictive accuracy in this sample. Regardless of the FGA findings, our results generally agreed with recent research advocating for ecologically valid balance assessments [16].

### 4.3. Limitations and Directions for Future Research

While our results suggest that balance assessments may be justified on a biannual basis, these results should be interpreted with some caution. First, our sample size for this pilot study was small and representative of a cohort with only mild-to-moderate PD severity with a smaller percentage of fallers than seen in previous balance assessment validity studies. In addition, a moderate number of participants were lost to follow-up at the six- and 12-month measurement points. Future research should examine larger samples of participants over a broader spectrum of disease severity and perhaps also consider different motor phenotypes within PD.

Second, we utilized previously established cut-off scores for all of the four balance assessments. These cut-off scores still resulted in false-negative and false-positive predictions. Since cut-off scores based on validity indices will likely change depending on the sample being studied, it is important to emphasize that cutoff scores should be utilized with caution and with the appreciation that any and all cutoff scores are simply guidelines and not definitive boundaries that separate fallers from nonfallers.

Third, our method of collecting fall incidence data, when used over a period of six months or more, can lead to an underreporting of falls [39]. As such, we suggest that future studies follow more rigorous procedures for collecting fall incidence data as outlined by Lamb and colleagues [40]. Future studies may also be designed to assess people with PD off anti-Parkinson medication to determine whether falls are more likely during this state.

## 5. Conclusion

Prospective identification of fall risk for individuals with PD is extremely important in order to demonstrate a need for therapeutic intervention aimed at reducing fall risk. Our comparison of varied duration of follow-up revealed that a six-month follow-up resulted in greater accuracy of fall prediction than a 12-month follow-up. In terms of accuracy of fall prediction during that six-month follow-up period, all tests provided moderate-to-strong accuracy for fall prediction with clinically meaningful alterations in the probability of being a faller. While the BESTest was slightly more accurate than the other tests, no test eliminated false-positive and false-negative predictions.

### 5.1. Rehabilitation Implications

None of the tests examined possesses acceptable predictive ability in determining who is at risk for falls within the next 12 months, suggesting the need for regular balance evaluations every six months among people with PD. Such a model of preventative evaluation and treatment twice per year is in keeping with other models of healthcare, such as the well-established system of prophylactic dental care in the United States. Such a model would likely be appropriate and beneficial to apply in the rehabilitative care of individuals with PD.

## Figures and Tables

**Table 1:** Demographics.

	6-Month Group (*n* = 51)	12-Month Group (*n* = 40)
Age	67.5 ± 8.8	67.3 ± 9.5
Years with diagnosis	7.7 ± 3.9	7.2 ± 4.1
UPDRS motor score	39.3 ± 13.3	37.8 ± 13.1
H&Y stages	2.4 ± 0.6	2.3 ± 0.6
Fallers (pretest probability of falling)	14/15 (0.275)	13/40 (0.325)

**Table:** (a) Predictive values at 6 months.

Balance measure	AUC (95% CI)	Score	Sensitivity	Specificity	LR + (95% CI)	LR − (95% CI)	Posttest probability with test ≤ cutoff value	Posttest probability with test > cutoff value
BESTest	0.89 (0.74–0.95)	≤69%	0.93	0.84	5.81 (3.69–9.14)	0.08 (0.04–0.17)	0.69	0.03
Mini-BESTest	0.87 (0.72–0.94)	≤20/32(63%)	0.86	0.78	3.97 (2.68–5.70)	0.18 (0.11–0.78)	0.60	0.07
BBS	0.87 (0.75–0.95)	≤47/56	0.79	0.86	5.64 (3.43–9.27)	0.24 (0.17–0.36)	0.68	0.09
FGA	0.80 (0.62–0.90)	≤15/30	0.64	0.81	3.37 (2.19–5.18)	0.44 (0.34–0.59)	0.56	0.15

**Table:** (b) Predictive values at 12 months.

Balance measure	AUC (95% CI)	Score	Sensitivity	Specificity	LR + (95% CI)	LR − (95% CI)	Posttest probability with test ≤ cutoff value	Posttest probability with test > cutoff value
BESTest	0.68 (0.45–0.83)	≤69%	0.46	0.74	1.77 (1.19–2.62)	0.73 (0.59–0.91)	0.46	0.26
Mini-BESTest	0.77 (0.55–0.89)	≤20/32(63%)	0.62	0.74	2.37 (1.66–3.34)	0.52 (0.39–0.68)	0.53	0.20
BBS	0.68 (0.45–0.82)	≤47/56	0.46	0.81	2.42 (1.53–3.82)	0.67 (0.54–0.82)	0.54	0.24
FGA	0.70 (0.50–0.83)	≤15/30	0.46	0.81	2.42 (1.53–3.82)	0.67 (0.54–0.82)	0.54	0.24

**Table 3:** *P* Values for 6 month NonInferiority test of AUC.

	BESTest	Mini-BESTest	BBS	FGA
BESTest	—	**0.02**	**0.04**	**<0.001**
Mini-BESTest	0.13	—	0.14	**0.001**
BBS	0.23	0.19	—	**0.01**
FGA	0.83	0.73	0.65	—

First variable in comparison is listed vertically, second variable is listed horizontally. Bold values indicate significant difference where first variable is superior to second variable.
